# Using infographics to improve trust in science: a randomized pilot test

**DOI:** 10.1186/s13104-021-05626-4

**Published:** 2021-05-29

**Authors:** Jon Agley, Yunyu Xiao, Esi E. Thompson, Lilian Golzarri-Arroyo

**Affiliations:** 1 Prevention Insights, Department of Applied Health Science, School of Public Health Bloomington, Indiana University Bloomington, Bloomington, IN USA; 2 Indiana University School of Social Work, Indiana University Bloomington and Indiana University-Purdue University Indianapolis, Bloomington, IN USA; 3 Indiana University Media School, Indiana University Bloomington, Bloomington, IN USA; 4 Biostatistics Consulting Center, School of Public Health Bloomington, Indiana University Bloomington, Bloomington, IN USA; 5 Present Address: 809 E. 9th St, Bloomington, IN 47405 USA

**Keywords:** Trust, Science, Science communication, Infographic, Pilot test

## Abstract

**Objective:**

This study describes the iterative process of selecting an infographic for use in a large, randomized trial related to trust in science, COVID-19 misinformation, and behavioral intentions for non-pharmaceutical prevenive behaviors. Five separate concepts were developed based on underlying subcomponents of ‘trust in science and scientists’ and were turned into infographics by media experts and digital artists. Study participants (n = 100) were recruited from Amazon’s Mechanical Turk and randomized to five different arms. Each arm viewed a different infographic and provided both quantitative (narrative believability scale and trust in science and scientists inventory) and qualitative data to assist the research team in identifying the infographic most likely to be successful in a larger study.

**Results:**

Data indicated that all infographics were perceived to be believable, with means ranging from 5.27 to 5.97 on a scale from one to seven. No iatrogenic outcomes were observed for within-group changes in trust in science. Given equivocal believability outcomes, and after examining confidence intervals for data on trust in science and then the qualitative responses, we selected infographic 3, which addressed issues of credibility and consensus by illustrating changing narratives on butter and margarine, as the best candidate for use in the full study.

**Supplementary Information:**

The online version contains supplementary material available at 10.1186/s13104-021-05626-4.

## Introduction

Misinformation about coronavirus disease 2019 (COVID-19) has spread widely, pervasively, and rapidly following the emergence of the disease [1-3]. The nature of this misinformation has ranged from clearly conspiratorial and misinformed, such as the idea that 5G cell towers spread COVID-19, to conceptually possible but implausible narratives about the origins of the disease and motivations underlying preventive public health efforts [4]. These narratives can spread very quickly [5] and have been associated, directly and indirectly, with harmful outcomes [6-8] as well as reduced personal wellness [9].

Prevention of COVID-19 misinformation uptake, as well as public health misinformation in general, is an important, but complex, area of research. For example, efforts to “fact check” or restrict access to misinformed narratives risk being counterproductive [10]. In addition, ethical concerns reasonably can be raised regarding attempts to restrict access to public speech. An alternative approach, often described as inoculation theory [11], focuses on interventions occurring prior to exposure to new misinformation. Such approaches have been used, for example, in addressing anti-vaccination narratives [12].

Based on recent studies [13, 14], our research team is currently investigating the potential for an intervention designed to improve public trust in science and scientists to serve as a possible approach for easily disseminated misinformation prophylaxis [15]. Specifically, we have proposed a randomized, controlled superiority trial comparing an infographic about the scientific process to a placebo infographic in terms of trust in science and scientists, reported believability of misinformed narratives about COVID-19, and behavioral intentions to engage in Centers for Disease Control and Prevention (CDC)-recommended prevention behaviors [15]. Part of the study protocol involves iterative design and selection of a single infographic from among multiple alternatives to be used in the primary trial. This Research Note describes the preliminary work and pilot test.

## Main text

### Infographic design

The infographics used in this pilot study were first conceptualized as text-only messaging based on our interpretation of underlying principles of trust in science as described by Nadelson et al. [16]. These included: (a) credibility and consensus, (b) epistemology, (c) trustworthiness, (d) stereotypes of scientists/“scientist-as-person,” and (e) science as methodology, not field. These ideas were workshopped extensively among the study team for clarity, and written descriptions of potential visual components were also recorded alongside each narrative.

As indicated in the protocol [15], these narratives were informally discussed within the authors’ nonscientific social networks. This feedback was discussed among the researchers and was used to make decisions about both the written and visual elements of the infographics. For example, non-scientists uniformly rejected statements beginning with “All scientists…” preferring instead the more guarded “Most scientists…” They also encouraged linking visuals to commonly discussed science, like the Space X program; we also observed that we should avoid politically controversial topics such as climate change in designing our infographics.

Our written descriptions were then presented in a meeting with a subcontracted graphics design team at Indiana University. That team prepared a set of five infographics and our research team reviewed the images, collectively made suggestions, and then the graphics design team modified the infographics accordingly (see Additional files 3, 4, 5, and 6). Though infographics had core themes, there was considerable overlap given the conceptual complexity of trust.

- Infographic 1: evolution in cigarette smoking recommendations (trustworthiness).
- Infographic 2: space X engineer putting on pants in the morning (scientist-as-person).
- Infographic 3: changing recommendations about butter/margarine (credibility/consensus).
- Infographic 4: John Snow and cholera (science as methodology).
- Infographic 5: relying on a weather forecast (epistemology).

### Pilot test methods

#### Data collection

The procedure for the pilot test was outlined in the published study protocol [15].

Data were obtained on December 19, 2020 from a sample of 100 US-based Amazon Mechanical Turk (mTurk) users ages 18 and older (individuals must be age 18+ to enroll as a mTurk worker). To ensure data quality, minimum qualifications were specified to initiate the survey (task approval rating > 99%, successful completion of more than 100, but fewer than 10,000 tasks, US-based IP address). Checks were embedded in the first part of the survey to control for dishonest workers, survey response bots or virtual private network users, and inattentive participants [17, 18]. Failing at these checkpoints resulted in the termination of the task and exclusion from the study, and participants were warned of this possibility on the study information page. Participants who successfully completed the study were compensated $0.61 USD. In the process of collecting 100 responses from workers, one additional worker refused consent, and 48 additional workers began the survey but were excluded prior to randomization for failing a quality check.

#### Procedures and instrument

Eligible workers completed the trust in science inventory, which consists of 21 Likert-type items yielding a mean score from 1 (low trust) to 5 (high trust) [16] and then were randomized with equal allocation to view one of the five infographics (*n *= 20 per arm, though due to simultaneous survey participation, infographic 4 had 21 workers and infographic 5 had 19). Participants were required to pause for at least one minute while viewing the infographic to loosely replicate uptake from multiple, but much shorter, exposures that would occur through social media. After viewing the infographic, workers were asked a qualitative question about the infographic’s meaning [19] and then were asked to complete a modified version of the narrative believability scale (nbs-12), which consists of 12 Likert-type items that produce a mean score from 1 (low believability) to 7 (high believability) [20]. Finally, workers completed the trust in science inventory a second time.

#### Analysis plan

Mean changes in trust in science between pretest and posttest were analyzed separately for each infographic using paired sample *t*-tests with unadjusted alpha set at .05. Differences in narrative believability between the five infographics were assessed using a one-way between-subjects analysis of variance (ANOVA), with Tukey’s HSD selected as a post-hoc test if the main effect was significant. All analyses were completed in SPSS v27.

Qualitative data were interpreted using a general inductive approach [21], with a primary focus on whether the participant described the infographic in such a way that it was clear that they understood the intended meaning.

### Pilot test results

#### Trust in science

The trust in science inventory was reliable at pretest (α = 0.940) and posttest (α = 0.946) for the full sample. The mean level of trust at pretest was 3.79 (*SD *= 0.67), and at posttest was 3.86 (*SD *= 0.66).

All study arms reported higher trust in science at posttest than at pretest (ranging from mean differences of 0.03 to 0.12, see Table 1), but only one within-arm difference was statistically significant (infographic 4, t(20)=− 2.11, *p *= 0.048, 95% CI of Diff: 0.001 to 0.239).

**Table 1:** Pretest-posttest comparison of trust in science scores

	Pretest			Posttest			Paired t-test (2-tailed)			
	Mean	SD	Range	Mean	SD	Range	Diff	95% CI	t	p
Overall (n = 100)	3.79	0.67	1.76–5.00	3.86	0.66	1.86–5.00	–	–	–	–
Infographic 1 (n = 20)	3.56	0.69	1.76–4.43	3.64	0.71	1.86–4.62	− 0.08	− 0.19 to 0.03	− 1.52	0.144
Infographic 2 (n = 20)	3.81	0.51	3.00–4.71	3.86	0.55	3.00–4.76	− 0.05	− 0.14 to 0.04	− 1.10	0.284
Infographic 3 (n = 20)	4.01	0.61	3.10–5.00	4.08	0.59	3.00–5.00	− 0.07	− 0.17 to 0.03	− 1.54	0.140
Infographic 4 (n = 21)	3.61	0.79	2.24–4.71	3.73	0.72	2.29–4.90	− 0.12	− 0.24 to -0.00	− 2.11	0.048
Infographic 5 (n = 19)	3.96	0.64	2.57–5.00	3.99	0.69	2.43–5.00	− 0.04	− 0.11 to 0.04	− 0.99	0.336

#### Narrative believability

The nbs-12 instrument was reliable for the full sample (α = 0.916). Each of the infographics had reasonably high believability, ranging from a low of 5.27 for infographic 1 to a high of 5.97 for infographic 2 (see Table 2). A one-way between-subjects ANOVA did not indicate any significant differences in mean narrative believability by infographic (*F*(4, 95) = 1.71, *p *= 0.154).

**Table 2:** Narrative believability scores

	Posttest		
	Mean	SD	Range
Overall (n = 100)	5.57	1.09	2.50–7.00
Infographic 1 (n = 20)	5.27	1.01	3.25–6.50
Infographic 2 (n = 20)	5.97	0.99	3.25–6.92
Infographic 3 (n = 20)	5.39	0.96	2.83–6.50
Infographic 4 (n = 21)	5.40	1.35	2.50–6.92
Infographic 5 (n = 19)	5.86	0.99	3.92–7.00

#### Qualitative results

The infographics were designed to convey specific meanings related to subconstructs of trust in science. Thus, responses where participants described the infographic in a way that reflected the message that we intended to communicate were marked as being ‘consistent,’ and those that shared other messages were marked as ‘inconsistent.’ In total, 25 of the 100 responses were determined to be inconsistent, split among: infographic 1 (*n *= 10), infographic 2 (*n *= 5), infographic 3 (*n *= 3), infographic 4 (*n *= 6), and infographic 5 (*n *= 1).

Exemplars of consistent and inconsistent responses, by infographic, are available in Table 3. In some cases, participants focused on the image itself rather than the meaning. For example, the intended emphasis of infographic 1 was on how doctors’ recommendations about cigarette smoking evolved due to new scientific evidence, but multiple people conflated scientists and doctors and focused on the medical recommendation rather than the reason for it. In other cases, it appeared that the narrative message was unclear, and some workers were not sure how to interpret an infographic. “I’m honestly not really sure what it was trying to communicate…” Finally, in a few cases, participants addressed the infographic’s meaning, but derived context or content outside of what we intended to communicate. This was observed most notably for infographic 4, which depicted John Snow and cholera: “The scientist came up with a theory and used a silly observation of what was seen to prove a theory, to which the scientific community agreed because of the scientist’s identity. Basically, it was an appeal to authority.” While the respondent identified the actors as scientists, they interpreted the message as implying that scientists must be trusted because they are scientists, and not because they have provided evidence to support their claim (which was the opposite of the intended message).

**Table 3:** Exemplars of qualitative data

Study arm	Inconsistent with intention	Consistent with intention
Infographic 1	The info AD I viewed on the last page described a doctor offering cigarettes in 1940, but condemning cigarettes in 2020. I could tell by the picture's response between the two characters	The infographic was trying to convey the message that scientists will alter their ways of thinking as new information becomes available, and can be trusted. It presented this message by showing how doctors in the 1940s did not understand the risks of smoking, but modern doctors advise patients not to smoke
Infographic 2	It displayed three vignettes and attempted to connect the ideas as a linear process. It showed a person thinking and theorizing, then working with others to test the theory which resulted in a rocket	The images showed that scientists spend a great deal of time thinking about what it is that they are trying to accomplish. But just like everyone else they get up in the morning and do the same things as us, like putting on our pants before work while thinking about how to accomplish launching a rocket. It's this commitment of time that results in an actual rocket launch
Infographic 3	Scientists have recommended that humans consume butter instead of margarine. A decade or two later, scientists changed their recommendation to giving priority to our using margarine due to the lower saturated fat content. More recently, science suggests that we use guidelines based on our own health needs for lower fat, trans fate, or a natural product with more saturated fat	Scientists make suggestions because of the findings they get from their research and they tend to make adjustments when they discover something contradicting the first suggestion because they are after the truth
Infographic 4	They were trying to find the cause for the cholera outbreak in London during the 1800s. They took samples of the water and looked under the microscope. They figured out that the water had bacteria in it and was contaminated. They all agreed on this finding	Even though scientists may be wrong sometimes, they are constantly questioning their findings. Just because someone has a hypothesis doesn't make that true. Scientists needs to be able to accept that they are not correct all the time and that will make them better in the end
Infographic 5	It is very important for parents to be able to communicate openly and effectively with their children. Open, effective communication benefits not only the children, but every member of the family. Relationships between parents and their children are greatly improved when there is effective communication taking place. In general, if communication between parents and their children is good, then their relationships are good as well	It was trying to communicate how the average person can benefit from listening to science/scientists and apply these benefits as they go about their daily life. In this case, a young boy wants to go out and play but on the screen they are telling there is a 90% chance of rain. So the adult in the screen is coming equipped with raincoats

The other 75 responses reflected at least partial understanding of our intended message without any additional unintended content (see Table 3).

## Pilot test discussion

The primary purpose of the pilot test was to assist our research team in selecting an infographic to use as part of a larger randomized trial [15]. As prespecified, no single analysis (change in trust, narrative believability) was to be interpreted as a sole means of determining which infographic to select for the upcoming trial. Further, the quantitative results were to be interpreted in tandem with the qualitative data.

### Quantitative

The pilot study was not powered to test for significant differences at pre- and post-test for the different infographics (and even then, within-group changes such as those we presented do not allow for inference of causality). Instead, our goal with those items was to check for any signs of potential iatrogenic changes (e.g., trust scores decreasing from pre- to post-test), which we did not observe, and to examine general trends.

The narrative believability score was not significantly different across arms—on a scale ranging from one to seven, the range of means was narrow, from 5.27 to 5.97, indicating generally good believability. Those scores, along with subscale variability (not shown, but available via the data and syntax), were conceptually consistent with other research on narrative believability [22]. Thus, no infographics were inherently eliminated from consideration due to the quantitative data alone.

### Qualitative

For multiple infographics (#1, 2, and 4), when respondents were asked to describe the meaning of the infographics in their own words, between 5 and 10 of participants veered away from the messages we were attempting to communicate. As a result, the qualitative evidence was weighted in support of infographics 3 and 5, for which most responses (17 and 18 descriptions, respectively) indicated that we successfully communicated our message (see Additional files 3, 4, 5, and 6).

On closer examination, though, we wondered whether the presence in infographic 5 of additional text, relative to other infographics, may have inflated the prevalence of consistent descriptions for that infographic. Because epistemology (the conceptual target of the infographic 5) is a complex concept, we felt that this extra text was necessary. However, some responses describing infographic 5 that were classified as ‘consistent’ contained direct restatements of provided text. As a result, it was unclear to us whether the high frequency of accurate restatement reflected an understanding of the message in the infographic or rote repetition of written text.

### Conclusions

The quantitative data didn’t strongly make the case for any specific infographic, and the infographic with arguably the “best” quantitative case (infographic 4) also appeared to create uncertainty and even oppositional interpretation. Infographics 3 and 5 both performed well qualitatively, but we were somewhat concerned that infographic 5’s qualitative performance may have been artificially high. As a result, we made the difficult decision to adopt infographic 3 for our larger study, though we note that a case could be made for infographics 4 and 5 as well, and encourage research and exploration of those files, which we have released alongside this note.

## Limitations

This cross-sectional pilot study was intended to select an infographic to be used as part of a larger randomized trial. As such, it was designed to provide exploratory insight into five infographics, but not to draw widely generalizable conclusions. Further, the breadth of infographics that we designed and tested was limited by our own belief that the messages should be trustworthy—that is, regardless of the goals of the study, our intention was that the messages communicated by the infographics should be things that are true, even if the possibility exists that exaggerated claims could produce larger effects.

## Supplementary Information

Additional files 3, 4, 5, and 6 are infographics used in this study. The concepts for each of the infographics were developed by the research team. The infographics were produced by Ms. Amanda Goehlert, a Designer on the Creative Team at Indiana University Studios. These images are considered part of this article for the purposes of licensing and use.

**Additional file 1:** COVID19 Pilot Test Data Raw.sav. Raw study dataset in SPSS (v. 27) format.

**Additional file 2:** Covid19 Pilot Test Syntax.sps. Analysis syntax used for the study (SPSS v. 27).

**Additional file 3:** Research illustrations_concept 1.jpg. Infographic 1 from Arm 1 of the study.

**Additional file 4:** Research illustrations_concept 2.jpg. Infographic 2 from Arm 2 of the study.

**Additional file 5: **Research illustrations_concept 4.jpg. Infographic 4 from Arm 4 of the study.

**Additional file 6: **Research illustrations_concept 5.jpg. Infographic 5 from Arm 5 of the study.

## Data Availability

Data generated during this pilot study as well as the analytic code are available as supplemental files alongside this manuscript (see Additional file 1* and *Additional file 2). All infographics except the one selected for use in the subsequent randomized controlled trial are also available as supplemental files (see Additional files 3, 4, 5, and 6).

## Abbreviations

ANOVA: Analysis of variance
CDC: Centers for Disease Control and Prevention
COVID-19: Coronavirus disease 2019
mTurk: Amazon Mechanical Turk
nbs-12: Narrative believability scale-12
