# Supplementary figures and images for: Using infographics to improve trust in science: a randomized pilot test
Source: BMC Res Notes. 2021 May 29;14:210. doi: 10.1186/s13104-021-05626-4 (PMC8164487; doi:10.1186/s13104-021-05626-4)

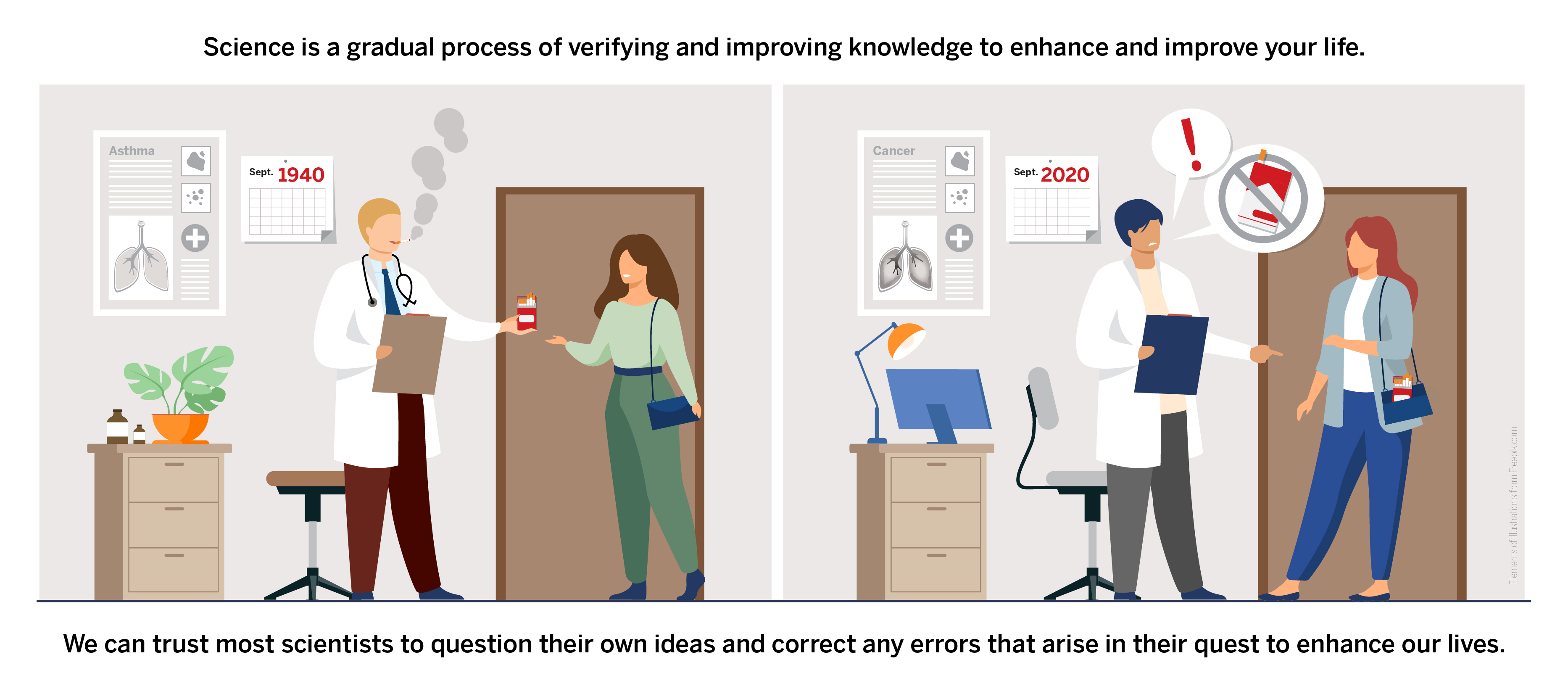

Supplement: Supplementary file 3 — Additional file 3: Research illustrations_concept 1.jpg. Infographic 1 from Arm 1 of the study. [file 13104_2021_5626_MOESM3_ESM.jpg]

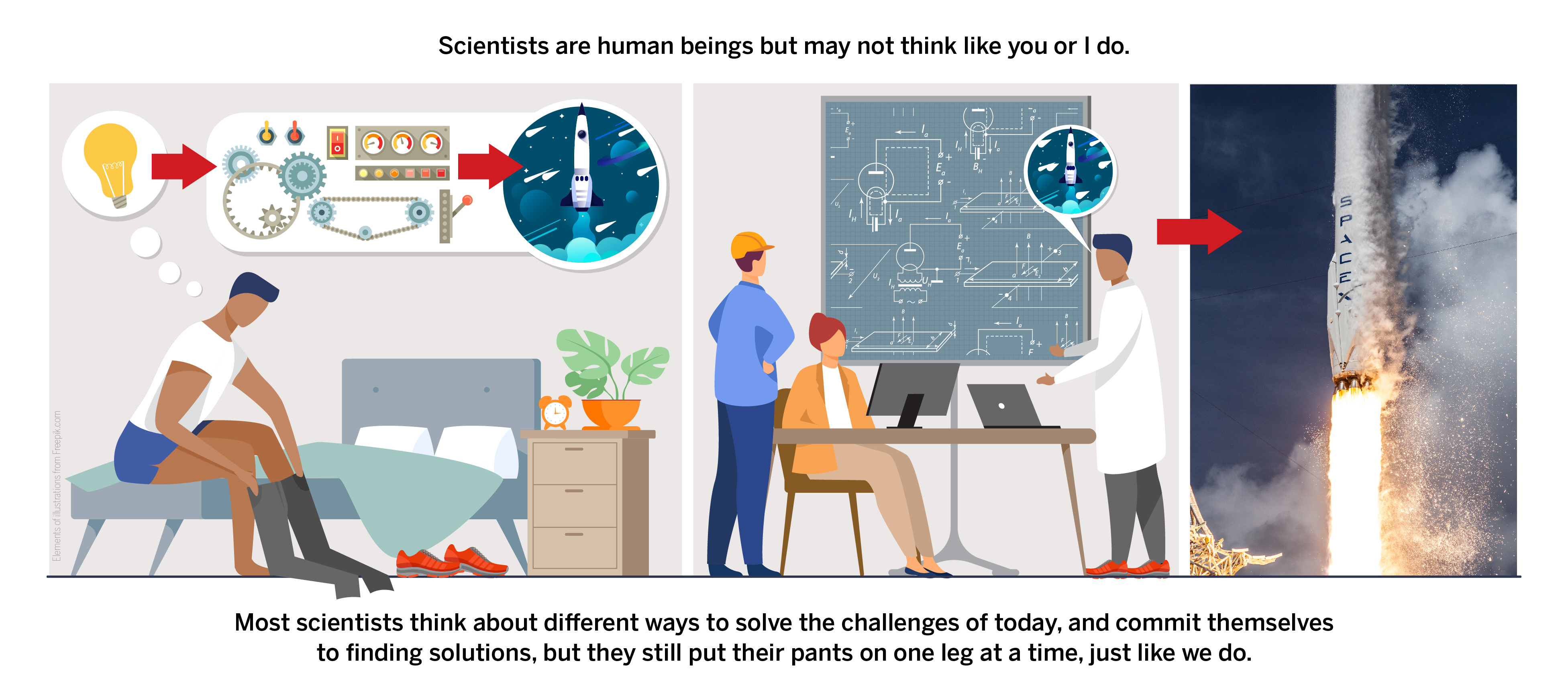

Supplement: Supplementary file 4 — Additional file 4: Research illustrations_concept 2.jpg. Infographic 2 from Arm 2 of the study. [file 13104_2021_5626_MOESM4_ESM.jpg]

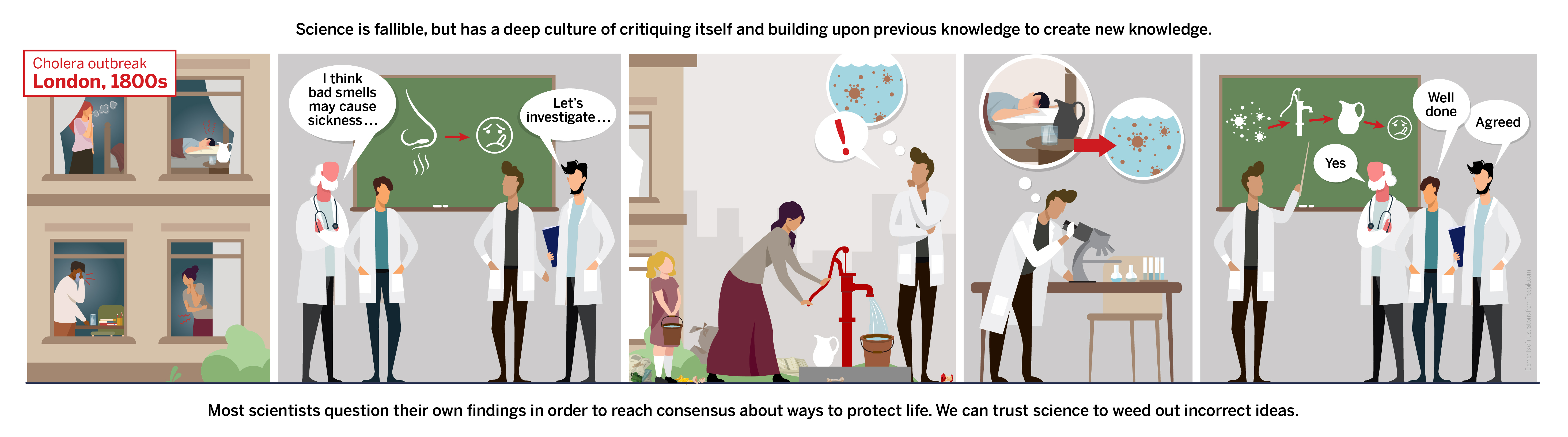

Supplement: Supplementary file 5 — Additional file 5: Research illustrations_concept 4.jpg. Infographic 4 from Arm 4 of the study. [file 13104_2021_5626_MOESM5_ESM.jpg]

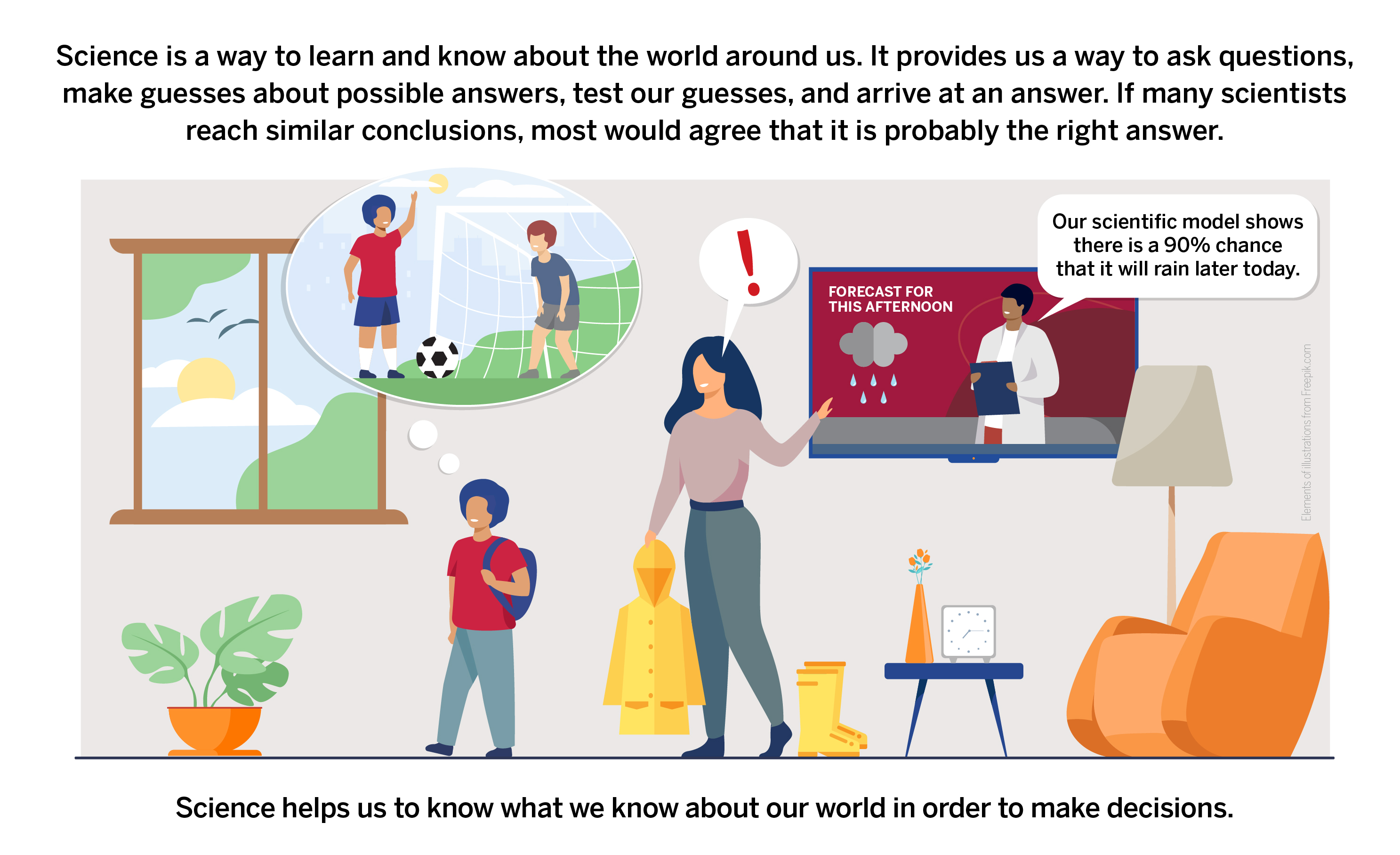

Supplement: Supplementary file 6 — Additional file 6: Research illustrations_concept 5.jpg. Infographic 5 from Arm 5 of the study. [file 13104_2021_5626_MOESM6_ESM.jpg]
